# Special Section Guest Editorial: Frontiers in Neurophotonics

**DOI:** 10.1117/1.NPh.11.1.014401

**Published:** 2024-03-28

**Authors:** Yves De Koninck, Paul De Koninck, Anna Devor, Flavie Lavoie-Cardinal

**Affiliations:** a CERVO Brain Research Centre, Québec City, Québec, Canada; b Université Laval, Department of Psychiatry and Neurosciences, Faculty of Medicine, Québec City, Québec, Canada; c Université Laval, Department of Biochemistry, Microbiology, and Bioinformatics, Faculty of Science and Engineering, Quebec City, Québec, Canada; d Boston University, Department of Biomedical Engineering, Boston, Massachusetts, United States; e Massachusetts General Hospital, Athinoula A. Martinos Center for Biomedical Imaging, Charlestown, Massachusetts, United States; f Université Laval, Institute Intelligence and Data, Québec City, Québec, Canada

## Abstract

The editorial presents the two-part Special Section on Frontiers in Neurophotonics.

We are pleased to present an exciting collection of papers under the umbrella of a two-part Special Section on Frontiers in Neurophotonics. This Special Section, appearing in *Neurophotonics* Volume 10 Issue 4 and Volume 11 Issue 1, was inspired by the Frontiers in Neurophotonics Symposium that was held in October 2022 in Québec City, Canada (Fig. 1). This symposium was the sixth in a series of international conferences dedicated to the new frontiers in microscopy and neuroscience, co-organized by Université de Bordeaux and Université Laval.

**Fig. 1:**
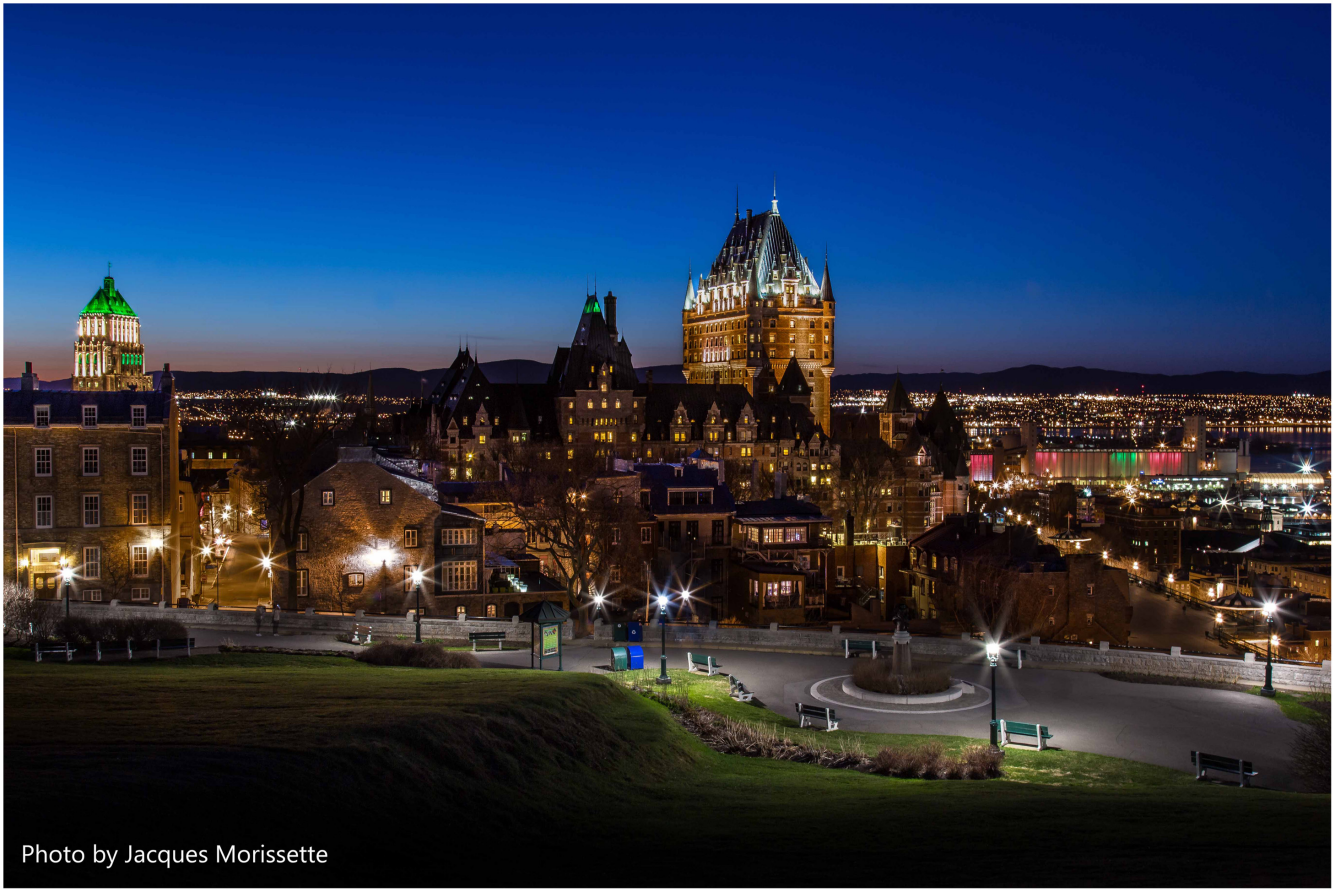
Québec City. Photo courtesy of Jacques Morissette.

The symposium brought together leading developers and users of advanced optical approaches to study brain functions--from receptor dynamics in synapses, to activity of neural circuits in intact brains, and the development of innovative approaches that can lead to new clinical and therapeutic opportunities. The present collection of papers reflects the broad scope of the meeting.

On the instrumentation side, we feature a primary research paper by Bancelin et al. on pushing super-resolution microscopy into imaging deeper inside the brain *in vivo.*^1^ With help of a spatial light modulator to overcome optical aberrations, the authors visualize dendritic spines in the hippocampus of a living mouse with an unprecedented level of detail. Another formidable challenge for *in vivo* imaging is the miniaturization of multiphoton microscopes to allow high-resolution, depth-resolved imaging of brain activity in freely moving animals, as outlined in a perspective article by Klioutchnikov and Kerr.^2^

A central focus of the Frontiers in Neurophotonics meeting is imaging of synaptic function. A primer by MacGillavry^3^ introduces the CRISPR/Cas9 genome editing method for fluorescent labeling of endogenous proteins such as postsynaptic density protein, 95 (PSD-95), for studies of synaptic plasticity. A perspective by Perrais et al. tackles the same topic with the use of pH-sensitive fluorescent proteins to visualize endo- and exocytosis of membrane receptors.^4^ In addition to imaging, manipulation of synaptic activity is critical for understanding of the underlying biological mechanisms. To that end, Caya-Bissonnette and Béïque discuss uncaging of glutamate and other photoactivation methods.^5^ These methods are paralleled by the development and improvement of tools for high-resolution imaging *ex vivo*, such as the multicolor super-resolution expansion microscopy—the focus of perspective article by Eilts et al.,^6^ which is featured on the cover of Volume 10 Issue 4.

The development of new and improved neurophotonic technologies necessitates new tools for data analysis and computational modeling. In this Special Section, we feature a primary research paper by Davoudi et al. that describes a computational model for correction of thermal fluorescence transients induced by ultrasound neuromodulation.^7^ In a perspective article by Bouchard et al., the authors discuss the challenges and possible mitigating strategies related to application of machine learning methods to optical datasets with insufficient amount of labeled data for efficient learning.^8^ Another perspective is focused on the problem of the association of pre- and postsynaptic proteins in super-resolution microscopy.^9^ Finally, combining neurophotonic data across individual labs and disjoint datasets often requires a standard coordinate system for mapping of neurons and projections. A perspective by Légaré et al. discusses this need in the context of sharing and integrating the larval zebrafish neurophotonic data.^10^

The rapidly expanding arsenal of neurophotonics tools enables novel biological applications. A review by Tanguay et al. summarizes the progress made in the development of biosensors for neurotransmitter noradrenaline that have given neuroscientists a novel view of the rodent brain during behavior.^11^ Another review by Foubert et al. focuses on the impact of calcium imaging in understanding the significance of astrocytic activity in the visual system development.^12^ Finally, a perspective by Doney et al. highlights the importance of super-resolution imaging and quantitative data analysis, originally developed for neuroscience applications, for imaging beyond the brain.^13^

Lastly, we worked together as a group to produce a summary paper^14^ reflecting the collaborative spirit of our community. In this “Community paper,” we talk about the symposium, as well as the Neurophotonics International Summer School, and focus on several of the topics discussed at the last event, including the work of Angela Getz and colleagues featured on the cover of Volume 11 Issue 1.
